# Glucagon-like peptide-1 receptor agonists in the perioperative period: Implications for the anaesthesiologist

**DOI:** 10.1097/EJA.0000000000001914

**Published:** 2024-02-02

**Authors:** Karim Gariani, Alessandro Putzu

**Affiliations:** From the Division of Endocrinology, Diabetes, Nutrition and Therapeutic Patient Education, Department of Medical Specialties, Geneva University Hospitals, Geneva, Switzerland (KG), Division of Anaesthesiology, Department of Anaesthesiology, Pharmacology, Intensive Care and Emergency Medicine, Geneva University Hospitals, Geneva, Switzerland (AP)

Editor,

Glucagon-like peptide-1 receptor agonists (GLP-1 RAs) are a well established pharmacological class of drug for the management of diabetes and sometimes as a treatment for patients who are overweight or obese without diabetes. GLP-1s are incretin hormones that are secreted by the gastrointestinal tract in response to nutrient intake.^1^ The actions include induction of insulin secretion, reduction of glucagon secretion, increasing satiety by targeting the hypothalamus, effecting reduced food intake, and delaying gastric emptying. All of the aforementioned actions contribute to the improvement of glucose control and weight-loss.

A total of seven GLP-1 RAs are currently approved, including six GLP-1 RAs that are administrated by subcutaneous injections (exenatide, exenatide-extended-release, liraglutide, lixisenatide, semaglutide, dulaglutide) and one GLP-1 RA taken orally (oral semaglutide) (Table 1). Furthermore, a dual agonist GLP-1/gastric inhibitory polypeptide (GIP) RA named tirzepatide has recently been approved in several countries for the treatment of diabetes and is being considered for approval for obesity and weight management.^2^

**Table 1:** Current glucagon-like petide-1 receptor agonists available

Name	Dosage	Route	Half-life
Lixisenatide (Lyxumia®)	10–20 μg once daily	SC	3–4 h
Exenatide (Byetta®)	5–10 μg twice daily	SC	2.4 h
Exenatide-extended-release (Bydureon®)	2 mg once weekly	SC	2.4 h
Semaglutide (Ozempic®)	0.25–1 mg once weekly	SC	160 h
Dulaglutide (Trulicity®)	0.75–1.5 mg once weekly	SC	90 h
Liraglutide (Victoza®)	0.6–1.8 mg once daily	SC	13 h
Semaglutide (Rybelsus®)	3–14 mg once daily	PO	7 days

PO, orally; SC, subcutaneous.

Beyond improvement of glucose control and weight reduction, GLP-1 RAs have been shown over large randomised clinical trials (RCTs) to significantly reduce major adverse cardiovascular events in diabetic patients, particularly among individuals with atherosclerotic disease or those considered to be at high-risk of cardiovascular disease.^3^

The exact prescription rate of GLP-1 RAs remains difficult to determine. However, the percentage of patients with type 2 diabetes treated with a GLP-1 RAs has increased from 3.2% to 10.7% from 2015 to 2020 in the United States, with yearly total prescription of more than 63 million.^4^ The prevalence of diabetes has significantly increased worldwide over the last few decades. In 2021, approximately 529 million people had diabetes, and this number is projected to reach 1.31 billion by 2050.^5^ Increasing rates of diabetes may consequently promote a rapid increase in the number of prescriptions for GLP-1 RAs in the coming years.

Common side effects reported with GLP-1 RAs are nausea, vomiting, and diarrhoea. These side effects are most often encountered during treatment initiation or after an increase in dosage. Initially, there was a suggested increased risk of acute pancreatitis and pancreatic cancer with GLP-1 RA use, but subsequent studies found no evidence for such risk.^6^

Analogously, initial studies suggested an increased risk of gallbladder issues. A meta-analysis of 76 RCTs found higher chances of these problems with GLP-1 RA use, especially for long durations, higher doses, and weight loss.^7^

GLP-1's effects on gastric emptying involve stimulating central nervous system GLP-1 receptors and brainstem vagal efferent fibres through the vagus nerve, possibly leading to slower emptying and raised intragastric volume.^1^

Delayed gastric emptying represents a potential anaesthetic concern due to the increased risk of pulmonary aspiration.^8^ Gastric emptying delay, independent of GLP-1 RA administration, is not rare in diabetic patients, as it is a common diabetes-related complication present in around 40% of cases of long-standing type 1 and 2 diabetes.^9^ Thus, the addition of GLP-1 RAs in patients with preexisting gastroparesis risks exacerbating this phenomenon.

Nondiabetic overweight and obese patients are theoretically at lower risk of preexisting gastroparesis compared to diabetic patients, regardless of GLP-1 RA use. Indeed, a study assessing 2.4 mg of semaglutide once weekly in obese subjects showed the absence of delayed gastric emptying after 20 weeks of therapy, assessed indirectly via paracetamol absorption, when compared with subjects taking a placebo.^10^

There is a growing number of case reports documenting episodes of perioperative pulmonary aspiration due to delayed gastric emptying in patients taking GLP-1 RAs. However, it remains uncertain if the GLP-1 RAs were the direct cause of pulmonary aspiration induced by increased gastric contents as long-standing diabetes was often present.

A retrospective study of 404 patients undergoing elective oesophagogastroduodenoscopies showed a significant increase in residual gastric contents in patients taking semaglutide for diabetes management or weight-loss when compared to control subjects. Of note, only one case of pulmonary aspiration was observed among all patients.^11^

A prospective study assessed the presence of solid contents with ultrasound in healthy volunteers recently starting semaglutide compared with a control group after 8 h fasting. The presence of intragastric solid contents was detected in 90% of individuals taking semaglutide vs. only 10% in the control group.^12^

Interestingly, several studies have specifically assessed the impact of GLP-1 RAs during the perioperative period in different types of surgery in terms of glycaemic control. A systematic review of RCTs found that GLP-1 treatment in surgical and critically ill patients reduces blood glucose levels and insulin administration without increasing hypoglycaemia episodes compared with control groups. However, these were only single-centre studies with small sample sizes; moreover, episodes of pulmonary aspiration were not reported.^13^

The perioperative management of GLP-1 RA drugs is discussed by some recent professional societies. In 2021, a consensus from the Society for Perioperative Assessment and Quality Improvement recommended discontinuing GLP-1 RAs on the day of surgery,^14^ while the British Centre for Perioperative Care recommended the continuation of the GLP-1 RA in the perioperative period.^15^

More recently, the American Society of Anesthesiologists published a press release on June 2023, providing detailed guidance on GLP-1 RA management in the perioperative period.^16^ According to the guidance, GLP-1 RAs should be paused on the day of the procedure for patients who take the medication daily and for one week for patients who take it weekly. Moreover, it advises consultation with an endocrinologist for patients using GLP-1 RAs for diabetes management. If the patient has not paused their GLP-1 RA medications, decision-making should be guided by gastric ultrasound. This may involve choosing between delaying the procedure, treating the patient as if they have a full stomach, or proceeding as usual. In an urgent scenario, full stomach precautions should be employed.

The issue of withholding these drugs is that, theoretically, three to five half-lives would be necessary to withhold to restore gastric motility, this could translate into more than 4 weeks in patients receiving long-acting GLP-1 RAs. However, withholding GLP-1 RAs for several days or weeks may not be an appropriate option due to the clear benefits of GLP-1 RAs for cardiovascular health and glucose control, in particular in diabetic patients. In addition, patients are often assessed by the anaesthesiologist during the preoperative consultation shortly before the procedure. Therefore, this approach could require postponing the surgery to allow for several weeks of GLP-1 RA cessation. Finally, after a prolonged period of GLP-1 RA discontinuation, it may be necessary to restart administration at the lowest dosage and respect the long titration protocol for this pharmacologic class to reduce or avoid side effects.

Future consideration should be given for a more individualised perioperative management plan for patients taking GLP-1 RAs, such as performing point-of-care gastric ultrasound.^17^ Strategies to mitigate the risk of aspiration should theoretically be considered, such as a strict liquid diet before surgery, the use of prokinetics treatment such as metoclopramide or erythromycin, and rapid sequence intubation. In higher risk patients a gastric decompression could be considered.

Anaesthesiologists should be aware of the potential increased risk of gastric aspiration in patients taking GLP-1 RAs due to delayed gastric emptying, even if relatively few events have been reported in the literature. The use of GLP-1 RAs may present a noteworthy aspect for inclusion in the perioperative risk evaluation and clinical strategy. Future large-scale studies are required to better estimate the risk of aspiration and develop an adapted perioperative assessment and management plan for patients taking GLP-1 RAs.
